# Annotations

**Published:** 1891-03-21

**Authors:** 


### ANNOTATIONS.

**The Suppression of Juvenile Smoking.**

Practical Manchester has made up its mind that boys must no longer be allowed to smoke. By permission of the Mayor a Conference was held in the Manchester Town Hall on Tuesday, and the aim of the promoters of the Conference was to ask the country to put an end to juvenile smoking, forthwith, by the double-barrelled method of school instruction and penal enactment. In a circular sent to us by Mr. R. Martin, M.D., the reasons for the suppression of juvenile smoking are clearly set forth, and they are such as commend themselves very emphatically to the medical mind, and doubtless also to the parental. Some of the facts given in the circular are decidedly startling to those who take a just view of the circumstances. It is stated, for example, that "During the last two years a sharp competition has arisen amongst the manufacturers of cheap cigarettes. Whereas, formerly, the lowest price of these articles was one penny each, now they can be bought at the rate of three for a penny, and in some cases two for a halfpenny. Owing to the lowness of the price, an increased number of the places where these cigarettes can be bought, together with the ease with which they can be carried about in the pockets of boys without attracting attention, it is not to be wondered at that the practice of smoking has become deplorably common amongst young lads, and has spread even to mere children." It is hardly necessary to say that with the disgusted feeling expressed by the circular in regard to these increased facilities for juvenile smoking, we entirely agree. The Manchester Conference proposes to make it penal for boys under a certain age to smoke at all. With that proposal also we are inclined to agree. But it seems to us that in punishing the silly lads whose immature brains lead them so disastrously astray, we should be doing a very small part of that which needs to be done. The worthless fellows who manufacture the cheap cigarettes for boys, and the equally contemptible creatures who retail them over the counter, ought first to be dealt with, and that very sharply. A stringent enactment imposing a fine so heavy as to make the trade in boys' cigarettes too unprofitable to be pursued is what is wanted. It must be borne in mind that boys do not stand on the same ground with regard to legislation as men. It is a serious thing to interfere with the liberty of the subject, when the subject is a grown-up man; and that is why temperance legislation presents so many thorny problems. But the case is quite different when youths and children are the subjects of proposed parliamentary enactments. It is to be hoped that the Manchester Conference, or any committee which it may appoint, will be able to draw up a prohibitory scheme which will commend itself to the judgment of doctors, parents, and legislators. Boys are given much more liberty in these days than they formerly were, and it is perhaps to their advantage that their freedom should be as large as possible. But when they use their liberty to poison themselves with tobacco, and to inflict a serious and permanent injury upon their health, it is time for the Legislature to put its foot down, and to effectually terminate such dangerous licence.

**Charity and Charity Organisation.**

In North Kensington there is what is called an Association of Friendly Workers amongst the Poor. The association consists of clergymen and ministers of religion of all denominations, of private persons of means and position, and of all who are anxious to furnish the workless poor with work, and the destitute with food. The Honorary Treasurer is Sir Roper Lethbridge, K.C.I.E., M.P., and the Honorary Secretary Mr. Donald Mackenzie. The association carries out its objects by visiting the poor at their own homes; and in order that the visiting may be done thoroughly, the North Kensington district is carefully mapped out, and there is assigned to each pair of workers just so many houses as they can visit regularly and keep themselves well informed about. In particular, it has been found that not only has destitution in Paddington been prevented by these means, but that every person of working age is supplied with profitable work of one kind or another. Now this seems to the writer, who is not a Paddingtonian, and who has no connection of any kind with the scheme, to be at least an unobjectionable method of doing necessary work. Perhaps a severely judicial attitude of mind would permit one to go even a little further, and to say that the North Kensington scheme is excellent in conception, and has proved to be efficient in practice. Of course, there is no reason why it should not be criticised. But a critic in the exercise of his functions necessarily makes two revelations: a revelation, from his point of view, of the thing criticised; but also a revelation of himself. This is exactly what a critic of the scheme has done in the February number of the *Charity Organisation Review*. The critic, in his wisdom, has declared that the "North Kensington, that is, the Paddington, Friendly Workers amongst the Poor . . . have entered upon a systematic invasion of every poor house in a specified district, with the object of obtaining particulars of the inhabitants, to be filed for confidential references. Some terrible figures are given in the report of the first year's work. Out of a total of 1,187 families only 217 refused information, as they felt themselves to be independent of extraneous aid. Did 970 then yield to the temptation to put themselves on the list of potential beggars? Or may we hope that in many instances a lingering sense of shame prompted the friendly worker to dissemble the purpose of the visit, and that the particulars were due to the wonderful courtesy of the poor and their limited range of conversation?" Here is the Charity Organisation Society, with its well-known methods of procedure, full blown and naked, but by no means ashamed. It would be impossible to condense into two or three sentences a completer description of that society and its methods than is here unintentionally given by its own exponents. In one sentence we may say that the Charity Organisation Society consists of an ounce of charity combined with many tons of criticism. The public, and particularly the public who work and do charitable deeds, need not care one straw for irresponsible criticism, whether it be adverse or favourable. The man who takes off his coat to work for charity, or who puts his hand into his pocket for the same object, feels that since he "pays the piper he is entitled to choose the tunes"; and if anybody pleases to stand by and sneer, the worker merely looks upon the scoffer as an impertinent fellow who has not been sufficiently birched at school. The Charity Organisation Society, indeed, seems to have fallen upon bad times. Theoretically, it is capable of rendering good service. But no organisation, however excellent its constitution, and however wise its aims, can do good work if it fall into the hands of inexperienced persons who apparently consider that the fittest ministers to conduct church services are the clown and the pantaloon.

**Hygiene and Demography.**

The forthcoming International Congress of Hygiene and Demography promises to be an event of some importance in the world of health. To our mind the most satisfactory thing about it is that it is an affair not exclusively or mainly of physicians, but of scientific and practical persons of all classes who take an interest in the many and important questions which bear upon the health of the community. It is beginning to be recognised and felt that health rather than wealth is the prime condition of national prosperity. Since the days of Adam Smith, political economists have had the ear of the people; and a very poor use they have made of their opportunities. The day of the physicians and physiologists has dawned. Shall we have any health prophets who will compel universal attention to their prophesyings? And if the next few generations should see a health bible written, will they turn the principles therein contained to practical account? We may all be thankful that so many men of rank, science, and learning are giving to health questions that public recognition and acknowledgment which their intrinsic importance demands. But we shall be grateful also if the conference brings home to the medical mind a full sense of its own duties and obligations in the matter of public health, and still more if it makes the public itself see that health and not wealth is the "dark horse" which is destined to win in the great world's races of the future.

